# ADAR1 overexpression is associated with cervical cancer progression and angiogenesis

**DOI:** 10.1186/s13000-017-0600-0

**Published:** 2017-01-21

**Authors:** Ying Chen, He Wang, Wenyi Lin, Ping Shuai

**Affiliations:** 1 Department of Obstetrics and Gynecology, West China Second University Hospital, Sichuan University, Chengdu, 610041 Sichuan China; 2 Department of Obstetrics and Gynecology, Sichuan Province, Chengdu Women’s and Children’s Central Hospital, Chengdu, 610091 Sichuan China; 3 Department of Pathology, Sichuan Province, Chengdu Women’s and Children’s Central Hospital, Chengdu, 610091 China; 4 Department Physical Examination, Sichuan People’s Hospital, Chengdu, 610072 Sichuan China

**Keywords:** Adenosine deaminase acting on RNA1, Cervical squamous cancer, Risk factor

## Abstract

**Background:**

This study aimed to assess the role of RNA-dependent adenosine deaminase (ADAR1) in cervical squamous cell carcinoma occurrence and progression.

**Methods:**

ADAR1 expression levels in stage IA and stage IIA cervical squamous cell carcinoma (group A), cervical intraepithelial neoplasia (CIN) specimens (group B), as well as normal and inflamed cervical tissue samples (group C) were assessed by immunohistochemistry. Clinical and pathological data of cervical squamous cell carcinoma patients undergoing surgery were retrospectively evaluated. Chi-square test, comparative analysis of survival curve, disease-free survival and COX risk assessment method were used to understand the association of ADAR1 with the occurrence and progression and prognostic significance of cervical squamous cell carcinoma.

**Results:**

ADAR1 is expressed in the cytoplasm and nuclei. The expression level was high in squamous cell carcinoma tissues (81.18%), while relatively low in the CIN group (21.56%). And there was no expression in non-cancerous tissues. The differences between them were statistically significant using *P* < 0.05 as criterion. One-factor analysis revealed that ADAR1 was significantly correlated with tumor diameter, horizontal diffusion diameter, vascular invasion, parametrial invasion, vaginal involvement, and pathologically diagnostic criteria for perineural invasion (PNI). Meanwhile, the overall survival rate of ADAR1 positive patients was significantly lower compared with that of patients with no ADAR1 expression (*P* < 0.05). Analysis also showed that disease-free survival time of ADAR1 positive patients was shorter than that of ADAR1 negative patients, and the difference was significant (*P* < 0.01). Finally, COX risk assessment showed that parametrical invasion had independent prognostic factors for overall survival of squamous cell carcinoma.

**Conclusions:**

Results indicated that ADAR1 might play an important role in the occurrence, progression and prognosis of cervical squamous cancer.

**Electronic supplementary material:**

The online version of this article (doi:10.1186/s13000-017-0600-0) contains supplementary material, which is available to authorized users.

## Background

Cervical carcinoma is the second most common malignant tumor in women around the world, and the first malignancy affecting females in developing countries. Indeed, about half a million women are diagnosed yearly with cervical cancer worldwide [1], with a mortality rate of 9%. In China, the number of new cases of cervical squamous cell carcinoma each year is 130,000, which accounts for 28% of newly diagnosed cases globally. And the mortality rate is 14%, which is higher compared with that obtained in other developing countries. For a long time, the studies of cancer mechanism had been greatly focused on the mechanism of DNA imbalance. Recently, we found that tumorigenesis might be caused by the process of RNA transcription, in which RNA editing enzyme played an important role. RNA editing is widespread in the evolutionary process [2]. It refers to RNA modifications and processing after the transcription of DNA into RNA. The edited RNA molecules may be translated to completely different proteins compared to their transcribed DNA sequences, hereby changing the genetic information [3–7]. Studies have shown that ADAR1 is a RNA-dependent adenosine deaminase, with highest expression in tumor cells. It mainly plays a role of RNA editing to change A into G in chronic myelocytic leukemia [8–10], thereby changing the normal physiological structures and functions of proteins. In addition, ADAR1 plays an important role in laryngeal squamous cell carcinoma, and tends to promote its occurrence and progression [11]. However, relevant studies on cervical squamous cell carcinoma have not been reported previously. In this study, The mRNA and protein expression levels of ADAR1 in cervical squamous cell carcinoma tissues, as well as their associations with patient prognosis, were assessed, in order to have a preliminary investigation of ADAR1’s role in cervical squamous cell carcinoma occurrence and progression.

### Materials and methods

#### Patients and clinical data

Tissue samples of patients were collected in Gynecology Department of Chengdu Women and Children’s Central Hospital. Between February 2012 and December 2015, 170 tissue samples from patients with stage IA-IIA cervical squamous cell carcinoma (Group A), 102 tissue specimens from patients with CIN (51 cases of CINI and and 51 cases of CIN II-III, respectively, Group B), as well as 31 normal and inflamed cervical tissue samples (Group C) were collected. Patients with cervical squamous cell carcinoma were selected according to the following critieria: (1) diagnosed with stage IA-IIA based on the FIGO (International Federation of Gynecology and Obstetrics) classification; (2) treated with radical hysterectomy and pelvic lymphadenectomy, with complete postoperative pathological data; (3) had complete postoperative follow-up data. All th patients were between 21–68 years old, with an average age of 48.79 years old. According to the FIGO classification, patients with stage I and stage IIA squamous cell carcinoma accounted for 68.24 and 31.76%, respectively. Clinical and pathological data of hospitalized patients were retrospectively analyzed.

## Methods

### Measurement and comparison of the expressions of ADAR1 in cervical squamous cell carcinoma in A, B, and C groups

Immunohistochemical paraffin sections came from the Chengdu Women and Children’s Central Hospital, Department of Pathology. All samples were fixed by 10% formaldehyde, embedded in paraffin, and treated with HE staining. And pathological results confirmed that to be cervical squamous cell carcinoma. All patients did not receive preoperative radiotherapy, chemotherapy and biotherapy.

ADAR1 rabbit anti-human monoclonal antibody was bought from abcom Company (Shanghai, China). Goat anti-mouse/anti-rabbit secondary antibody reagents were purchased from Guangzhou Shenda Biological Products Technology Co., Ltd (Guangzhou, China). Procedures exactly followed the manufactures’ instructions:a) Paraffin section was baked at 65 °C for 3 h, immersed in xylene to dewax for l0 min for two times, and then dehydrated using gradient alcohol; b) Paraffin section was soaked in 3% hydrogen peroxide for l0 min, and then washed three times using PBS buffer, 3 min for each time; c) Microwave antigen repair was performed using 0.4 g citric acid/3 g sodium citrate mixed solution (1 L.pH 6.0). After 30 min of natural cooling, paraffin section was washed two times using PBS buffer, 3 min each time. d) The first antibody (ADAR1 rabbit anti-human monoclonal antibody) was added and then paraffin was placed in a humid chamber incubator at 37 °C for 60 min, following with 3 times of wash using PBS buffer; e) The secondary antibody, i.e. goat anti-mouse/anti-rabbit, was added and then paraffin was incubated at room temperature for 30 min before PBS buffer washing for 3 times; f) DAB staining at room temperature for 5 min, and then washed using tap water for 15–20 min; nuclei staining using hematoxylin for 2–3 min, and then washed using tap water for half an hour; g) 85% alcohol, 90% alcohol, 95% Ethanol, pure ethanol wash for 5 min × 1 times, xylene wash for 5 min × 2 times. After all of these steps, check the paraffin using microscope. By the way, we set positive control and negative control: normal cervical tissue as a negative control with PBS solution instead of primary antibody; known esophageal squamous cell carcinoma as ADAR1 positive control.

Immunohistochemistry was used to detect whether Pathologically diagnostic criteria for perineural invasion (PNI) was expressed in cervical squamous cell carcinoma. Pathologically diagnostic criteria for perineural invasion (PNI) refers to a focal invasion and metastasis phenomenon, with tumor cells entering any layer of epineurium, perineurium or endoneurium along nerves fibers or around the nerves, or aggregating and wrapping the nerves (≥33% of nerve circumference) as well as extending along them [12].

Sample collection methods were the same as above, with the only difference was to use S-100 rabbit anti-human monoclonal antibody instead (purchased from Guangzhou Shenda Biological Products Technology Co., Ltd, Guangzhou, China).

### Statistical analysis of association of ADAR1 with clinical pathology of cervical squamous cell carcinoma

According to FIGO staging of cervical pathology indicators in combination with clinical practice, we chose 15 factors to describe the progression of cervical squamous cancer, including menopause, motherhood, clinical stage, keratosis, tumor size, horizontal diffusion distance, stromal invasion, histological grade, vascular invasion, parametrial margin, parametrial invasion, vaginal involvement, lymph node metastasis, HPV infection, and perineural invasion. They were compared with ADAR1 expression to explore the association of ADAR1 with progress of cervical squamous cancer. Clinical and pathological data of group A undergoing surgery were retrospectively evaluated, using chi-square test to verify the association of ADAR1 with the occurrence and progression of cervical squamous cell carcinoma. Comparative analysis of survival curve, disease-free survival and COX risk assessment method were performed to verify the prognostic significance of ADAR1 in cervical squamous cell carcinoma.

### Follow-up

Mainly, patients with cervical squamous cell carcinoma were followed-up by telephone, mails or postoperative visits, from pathological diagnosis to December 2015. Follow-up lasted for 1–47 months, with an average follow-up time of 30.4 months.

### Immunohistochemical assessment

Positive signals appeared as brown staining in the nuclei and cytoplasm and percentage of positive cells/total cell count is used to measure the expression level of ADAR1. With the normal squamous epithelium as a reference, positive ADAR1 expression was indicated by a higher expression, and negative expression, refers to weaker or comparable expression levels, compared with the amounts obtained for the corresponding squamous epithelium specimens. The following scales were used for staining degrees: (−), only basal cells stained, with positive cell rate <5%; (+), focal lesion stained, with positive cell rate ranging from 5 to 30%; (++), extensive staining with positive cell rate of 31- 60%; (+++), diffused staining with positive cell rate greater than 60%. Escaping slices or those with very small numbers of tumor cells (cell count < 200) were considered ineffective (18 slices).

### Statistical analysis

Statistical analyses were performed using the SPSS 19.0 software. One-factor analysis of categorical variables was conducted with chi-square test; survival curves were generated by the Kaplan-Meier method. The parameters of statistical significance from univariate chi-square test were included in the COX risk assessment to test whether ADAR1 was an independent prognostic factor for overall survival in cervical squamous cell carcinoma.

## Results

### Immunohistochemical data analysis

ADAR1 is expressed in the cytoplasm and nuclei. Negative and weakly positive signals were considered to be normal expression, while moderately and strongly positive signals were considered to be high expression, i.e. positive expression. Interestingly, the positive rate was found to be 81.18% (140/170) for Group A and 21.56% (22/102) for Group B, while all Group C cases showed no expression. In addition, the signal intensity gradually increased with the lesion severity. The differences among these groups were statistically significant (*p* < 0.05).

**Fig. 1:**
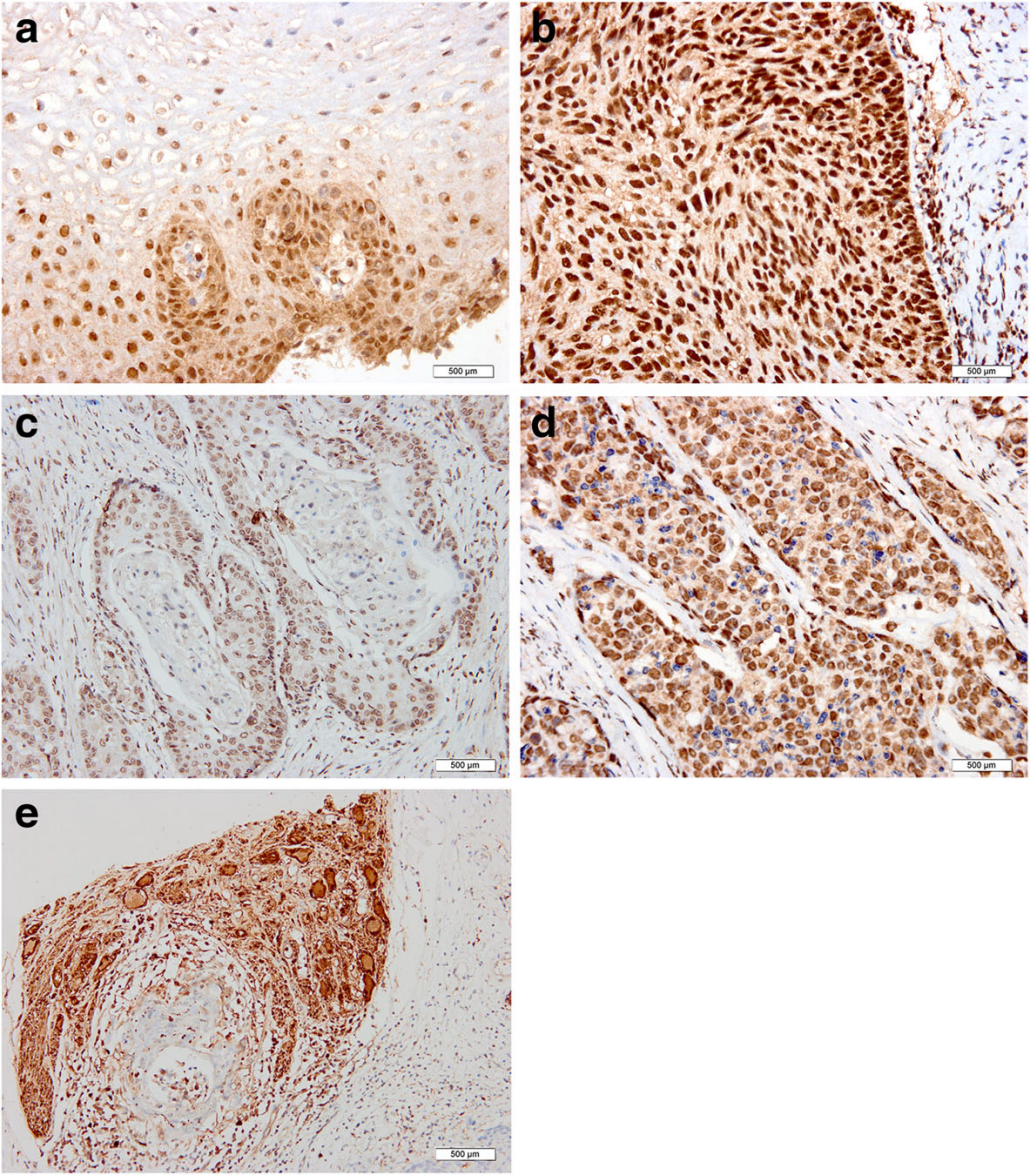
Immunohistochemistry (IHC) data showing ADAR1 expression levels in normal cervical, CIN, and cervical squamous cancer tissue specimens. **a** Normal squamous epithelium (IHC × 200); **b** Uterine neck CIN III (IHC × 200); **c** Cervical keratinizing squamous cell carcinoma (IHC × 200); **d** Cervicalnon-keratinizing squamous cell carcinoma (IHC × 200). **e** Perineural invasion exhibited messy and irregular next to cervical squamous cell carcinoma lesion (IHC × 200)

In ADAR1 positive tissue of cervical squamous cell carcinoma, the PNI was found around the foci, accounting for 14.71% (25/170), which was mainly for the nerve fibers distributing in the cancer stroma irregularly, and perineural invasion around the foci was visible with irregular growth of nerve and messy. However, the PNI expression in ADAR1 negative tissue was negative. The difference was statistically significant (*p* < 0.05) (Fig. 1).

### Associations of ADAR1 and cervical squamous cell carcinoma related factors

One-factor analysis data of ADAR1 are shown in Table 1. A total of 15 factors were analyzed, including menopause, gravidity, clinical stage, keratinization, tumor diameter, horizontal diffusion diameter, stroma invasion depth, histological grade, vascular invasion, parametrial margin, uterine invasion, vagina involvement, lymph node metastasis, HPV infection, and PNI. Interestingly, tumor diameter, horizontal diffusion diameter, vagina involvement, vascular invasion, uterine invasion and PNI were significantly associated with ADAR1 (*p* < 0.05), as high-risk factors affecting ADAR1 expression. Besides uterine invasion which had a *p* < 0.05, the remaining factors showed *p* < 0.01, indicating statistical significance (Table 1).

**Table 1:** One-factor analysis of ADAR1

Characteristics	Case (*n* = 170)	Negative	Positive	*P*
Menopause				0.144
Yes	68	16(38.23%)	52(76.47%)	
No	102	15(14.71%)	87(85.29%)	
Gravidity				0.212
< 3	36	4(11.11%)	32(88.89%)	
≥ 3	134	27(20.15%)	107(79.85%)	
Clinical stage				0.224
I	116	24(20.69%)	92(79.31%)	
IIA	54	7(12.96%)	47(87.04%)	
Tumor diameter				0.000**
≤ 4 cm	96	27(28.13%)	69(71.88%)	
> 4 cm	74	4(5.41%)	70(94.59%)	
Keratinization				0.079
Yes	90	12(13.33%)	78(86.67%)	
No	80	19(23.75%)	61(76.25%)	
Horizontal diffusion diameter				0.000**
≤ 4 cm	102	28(27.45%)	74(72.55%)	
> 4 cm	68	3(4.41%)	65(95.59%)	
Stroma invasion				0.612
Superficial stroma	8	2(25.00%)	6(75.00%)	
Deep stroma	162	29(17.90%)	133(82.10%)	
Vascular invasion				0.008*
Negative	36	12(33.33%)	24(66.67%)	
Positive	134	19(14.18%)	115(85.82%)	
Lymph node metastasis				0.076
Negative	54	14(25.93%)	40(74.07%)	
Positive	116	17(88.73%)	99(85.34%)	
Parametrial invasion				0.023*
Negative	126	28(22.22%)	98(77.78%)	
Positive	44	3(6.82%)	41(93.18%)	
Parametrial margin				0.855
Negative	152	28(18.42%)	124(81.58%)	
Positive	18	3(16.67%)	15(83.33%)	
Vagina margin				0.001**
Negative	80	23(28.75%)	57(71.25%)	
Positive	90	8(88.89%)	82(91.11%)	
Histological grade				0.318
G1	30	6(20.00%)	24(80.00%)	
G2	52	6(11.54%)	46(88.46%)	
G3	88	19(21.59%)	69(78.41%)	
HPV infection				0.955
Negative	16	3(18.75%)	13(81.25%)	
Positive	154	28(18.18%)	126(81.82%)	
PNI				0.011*
Negative	31	31(100.00%)	0(0.00%)	
Positive	139	114(82.01%)	25(17.99%)	

*p < 0.05, **p < 0.01

### Effect of ADAR1expression in cervical carcinoma prognosis

**Fig. 2:**
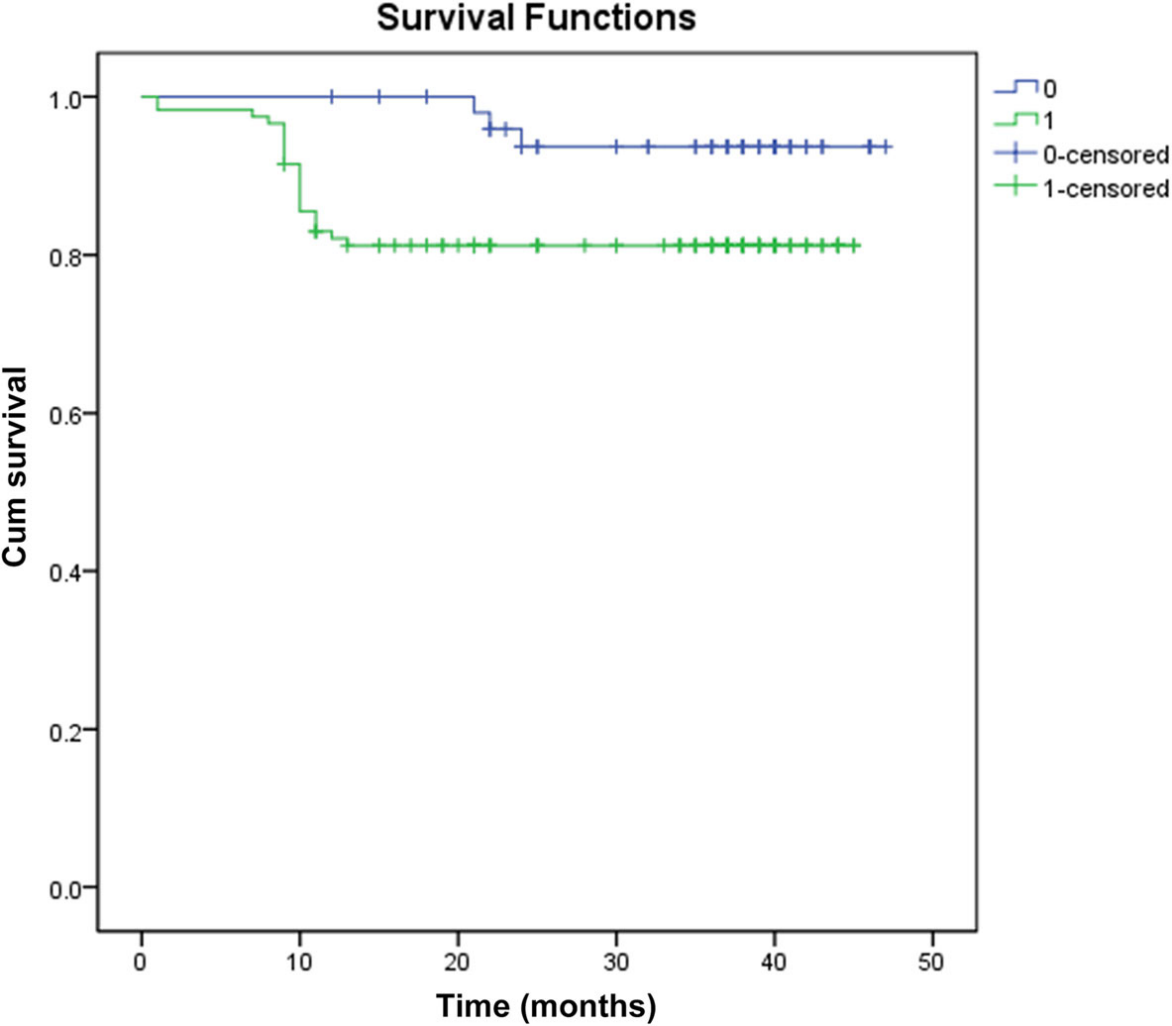
Overall survival curve of patients with ADAR1 positive and ADAR1 negative cervical squamous cell carcinoma. Survival time of ADAR1 positive group was 38.252 ± 1. 301 month, survival time of ADAR1 negative group was 45.458 ± 0. 863 months. The difference was statistically significant (*χ*2 = 5.101, *p* = 0.024). 0 = ADAR1 negative group; 1 = ADAR1 positive group

**Fig. 3:**
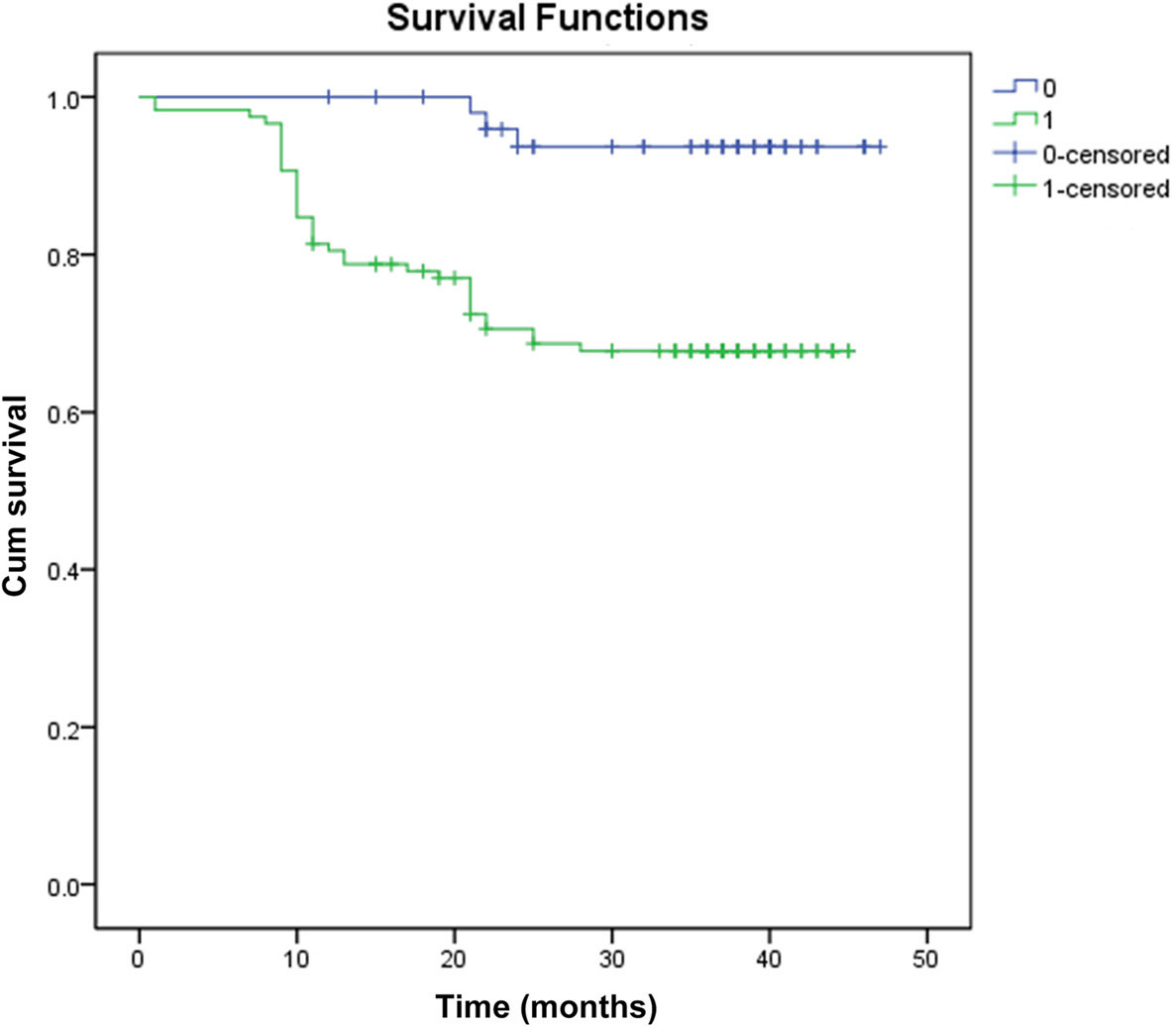
Disease-free survival curve of patients with ADAR1 positive and ADAR1 negative cervical squamous cell carcinoma. Overall disease-free survival time was 39.048 ± 1.118 months. Disease-free survival time of ADAR1 negative group was 45.458 ± 0.863 months, disease-free survival time of ADAR1 positive group was 34.877 ± 1.408. The difference was statistically significant (*χ*2 = 12.925, *p* < 0.001). 0 = ADAR1 negative group; 1 = ADAR1positive group

Overall survival times of ADAR1 positive and negative patients were 38.25 ± 1.3 and 45.46 ± 0.86 months, respectively, with a significant difference of *p* < 0.05 (*χ* ^2^ = 5.101, *p* = 0.024) (Fig. 2). The overall disease-free survival times were 39.048 ± 1.118 months. The disease-free survival times of ADAR1 negative and positive patients were 45.458 ± 0.863 and 34.877 ± 1.408 months, respectively, with a significant difference (*χ*2 = 12.925, *p* = 0.000) (Fig. 3). From overall survival curve, we could see that the 1-year and 2 year survival rates for ADAR1 negative group were 100 and 93.8%, respectively; while the 1 year and two survival rates for ADAR1 positive group were 82.1 and 81.2%, respectively. From disease-free survival curve, we could see that 1-year and 2 year survival rates for ADAR1 negative group were both 100%; the 1-year and 2 year disease-free survival rates for ADAR1 positive group were 80.5 and 70.6%, respectively. Taken together, our data suggest that ADAR1 positive patients have worse prognosis. To further study the effect of ADAR1 positive expression on overall survival rate of cervical squamous cell carcinoma, we used multivariate COX proportional hazard model, and it is showed that parametrial invasion for overall survival of ADAR1 positive cervical squamous cell carcinoma had an independent prognostic factor with statistical significance (Table 2).

**Table 2:** Multivariate analysis of the predictive value of cervical squamous cell carcinoma overall survival for each variable clinical case

Variables	B	S.E.	Wald	df	Sig.	Exp(B)	95% CIfor EXP (B)	
							Lower	Upper
PNI	-.295	.634	.217	1	.641	.744	.215	2.579
Tumor diameter	.407	.454	0820	1	.370	.666	.212	2.969
Horizontal diffusion	-.231	.673	.118	1	.732	.794	1.183	15.581
Vessel invasion	.016	.572	.001	1	.978	1.016	.331	3.115
Parametrial invasion	1.090	.401	7.411	1	.006*	2.976	1.357	6.525
Vagina involvement	-.173	.574	.091	1	.763	.841	.273	2.590
Constant	-.277	1.039	.071	1	.790	.758		

*p < 0.05

*PNI* pathologically diagnostic criteria for perineural invasion

*B* coefficient of regression

*S.E* standard error

*Wald* Wald Chi-Square

*df* degree of freedom

*sig* statistically significant

*EXP(B)* B coefficient index

*95% CI for EXP (B)* 95% confidence interval

*OR* odds ratio

## Discussion

ADAR1, also known as RNA editase, has attracted increasing attention in recent years. Athanasiadis et al. [13] found that ADAR1 had anti-viral and anti-tumor effect, which was due to the fact that ADAR has a Z-DNA-binding domain, zalpha, differing with other members of the ADAR family. The Z-DNA-binding domain could bind to the CPG sequence with left-handed helical structure with a high affinity and specificity. Once bound, it is associated with interferon response, leading to the anti-tumor effect. On the other hand, its erroneous editing or absence of editing may be closely associated with the occurrence of tumors. The possible mechanism may be the alteration of proteins involved in important pathways, thereby leading to tumor occurrence and progression. Leilei et al. [14] found by transcriptome sequencing that ADAR1 with A-to-I RNA editing might be a potential driver in the pathogenesis of human cancer, especially liver cancer. Jochen et al. [15] found that ADAR1 editing for nuclear adenosine of nerve tissue was crucial for embryonic development of mouse liver. They generated inducible ADAR1 interference in mice and found that ADAR1 played an important role in the maintenance of adult hematopoietic stem cells (HSC) and the inhibition of interferon signaling pathway. The interferon signaling pathway can protect many pathological processes of the body mainly by downregulating the activation of harmful effect on interferon, avoiding chronic inflammation, autoimmune diseases and cancer [16, 17]. It is also known that ADAR1 shows different expression levels in cancer tissues such as laryngeal cancer, bladder cancer, and hematologic malignancies, as well as different stages of tumor progression. However, the molecular mechanisms underlying its effects are largely unclear, with no report linking ADAR1 to cervical squamous cell carcinoma.

### ADAR1 expression in different cervical tissues

As shown above, we found that ADAR1 was highly expressed in the cytoplasm and nuclei and its expression level gradually increased with cervical disease stage. The close association of ADAR1 with cervical squamous cell carcinoma, as well as its progress indicates it may play an oncogenic role in the occurrence and progression of cervical squamous cell carcinoma. Thus, ADAR1 might be considered as an oncogene in cervical squamous cell carcinoma.

### Associations of ADAR1 with different clinicopathologic features of cervical squamous cell carcinoma

A prospective study on extensive hysterectomy for initial treatment of stage 1B cervical carcinoma conducted by the [gynecologic oncology group] GOG revealed that tumor diameter, invasion depth and vascular invasion are independent prognostic factors [18]. Except this,as shown above, we found that horizontal diffusion diameter, parametrial invasion, and vagina involvement were also significantly associated with ADAR1 expression. Surgery for phase Ib–IIa cervical carcinoma with a diameter greater than 4 cm is very difficult and prone to postoperative focal recurrence and distant metastasis [19]. This is especially true when a large tumor size is combined with deep myometrial invasion. Based on the GOG study, it indicates that tumor diameter, invasion depth and vascular invasion are related to horizontal diffusion diameter, parametrial invasion, and vagina involvement, affecting prognosis. Our findings indicated an association of ADAR1 with the metastasis, invasion, and malignancy of cervical squamous cell carcinoma. However, the mechanism is not clear, which needs for further study.

Previous studies reported that PNI is a risk factor predicting tumor recurrence and death, and tumor invasion, whether to nerve trunks or endings, could increase the risk of recurrence and decrease survival [20–22].

In this study, we also found that PNI existed in the foci of ADAR1 positive cervical squamous cell carcinoma, indicating the presence of the nerve fibers might play a role in regulating the progress of cervical squamous cell carcinoma. Furthermore, it was found that the ADAR1 positive cases were concurrent with PNI expression, indicating that PNI was associated with ADAR1 positively. These data suggest that ADAR1 is an important indicator of prognosis in cervical squamous cell carcinoma.

### Relationship between ADAR1 and cervical squamous cell carcinoma prognosis

From the overall survival curve, there was difference in survival rate within 24 months between the ADAR1 positive group and the ADAR1 negative groups, and the two sets of data tended to balance in 30 months. The similar results were also found in disease-free survival curve. From the figure, the recurrence and mortality of the stage I-IIA patients with cervix squamous cell carcinoma in 30 months after surgery were greatly reduced with stabilized condition. Combined with result of Cox proportional hazards assessment and the PNI expression in squamous cell carcinoma, it suggested that ADAR1 expression was not only associated with the metastasis, invasion, and malignancy of cervical squamous cell carcinoma but also closely related to the prognosis, which further confirmed that ADAR1 expression had guiding significance for prognosis of cervical squamous cell carcinoma.

## Conclusion

Our preliminary study demonstrates the increased expression of ADAR1 is associated with development and progress of cervical squamous cell carcinoma. However, further studies are warranted to investigate its underlying molecular mechanisms.

## Additional file

Additional file 1: Original data of baseline, clinical pathological diagnosis, prognosis and follow-up for ADAR1 patients. (DOC 825 kb)

## Abbreviations

ADAR1: Adenosine deaminase
CIN: Cervical intraepithelial neoplasia
HSC: Hematopoietic stem cells
