# Supplementary material for: ADAR1 overexpression is associated with cervical cancer progression and angiogenesis
Source: Diagn Pathol. 2017 Jan 21;12:12. doi: 10.1186/s13000-017-0600-0 (PMC5251241; doi:10.1186/s13000-017-0600-0)
Supplement: Additional file 1: — Original data of baseline, clinical pathological diagnosis, prognosis and follow-up for ADAR1 patients. (DOC 825 kb) [file 13000_2017_600_MOESM1_ESM.doc]

| 姓名 | 年龄分段 | 年龄 | 绝经与否 | 孕产次(统计学) | 孕产次 | 术前化疗 | 临床分期 | 手术名称 |
| --- | --- | --- | --- | --- | --- | --- | --- | --- |
| name | age group | age | menopause  or not | Gravidity and  parity history | Gravidity and  parity history | Preoperative chemotherapy | clinical stages | Operation |
|  | 20-30=1 | | No=0 | ≤3=0 |  |  | Phase I=1 | Cervical cone resection=1 |
|  | 31-40=2 |  | Yes=1 | ＞3=1 |  |  | Phase II=2 | Total hysterectomy =2 |
|  | 41-50=3 |  |  |  |  |  |  | Wide + basin clear +  retain one or both ovaries=3 |
|  | 51-60=4 |  |  |  |  |  |  | Wide + pelvic clear +  bilateral attachment  resection=4 |
|  | 61-70=5 |  |  |  |  |  |  |  |
| name1 | 3 | 43 | 0 | 1 | G3P2 | 0 | 2 | 3 |
| name2 | 4 | 53 | 1 | 1 | G3P3 | 0 | 1 | 4 |
| name3 | 4 | 53 | 1 | 1 | G3P3 | 0 | 2 | 4 |
| name4 | 2 | 39 | 0 | 1 | G3P2 | 0 | 1 | 3 |
| name5 | 4 | 56 | 1 | 1 | G7P3 | 0 | 1 | 4 |
| name6 | 4 | 60 | 1 | 0 | G2P1 | 0 | 1 | 4 |
| name7 | 3 | 44 | 0 | 1 | G6P2 | 0 | 1 | 3 |
| name8 | 3 | 46 | 0 | 0 | G2P1 | 0 | 1 | 4 |
| name9 | 4 | 51 | 0 | 1 | G5P2 | 0 | 1 | 4 |
| name10 | 3 | 45 | 0 | 1 | G3P1 | 0 | 1 | 4 |
| name11 | 4 | 56 | 0 | 0 | G2P1 | 0 | 1 | 4 |
| name12 | 3 | 43 | 0 | 0 | G2P1 | 0 | 1 | 4 |
| name13 | 3 | 44 | 0 | 1 | G3P1 | 0 | 1 | 4 |
| name14 | 4 | 56 | 1 | 1 | G3P1 | 0 | 2 | 4 |
| name15 | 2 | 36 | 0 | 1 | G3P1 | 0 | 2 | 3 |
| name16 | 4 | 58 | 1 | 1 | G4P2 | 0 | 1 | 4 |
| name17 | 4 | 52 | 1 | 1 | G4P1 | 0 | 2 | 4 |
| name18 | 2 | 40 | 0 | 0 | G1P1 | 0 | 2 | 3 |
| name19 | 2 | 39 | 0 | 0 | G2P1 | 0 | 1 | 3 |
| name20 | 4 | 54 | 1 | 1 | G4P3 | 0 | 1 | 4 |
| name21 | 4 | 59 | 1 | 1 | G3P3 | 0 | 1 | 4 |
| name22 | 2 | 35 | 0 | 1 | G6P2 | 0 | 1 | 3 |
| name23 | 4 | 57 | 1 | 1 | G8P2 | 0 | 2 | 4 |
| name24 | 4 | 56 | 1 | 0 | G2P1 | 0 | 1 | 4 |
| name25 | 3 | 42 | 0 | 1 | G3P1 | 0 | 1 | 3 |
| name26 | 4 | 51 | 1 | 1 | G6P2 | 0 | 1 | 4 |
| name27 | 3 | 44 | 0 | 1 | G6P3 | 0 | 1 | 4 |
| name28 | 4 | 55 | 1 | 1 | G4P4 | 0 | 2 | 4 |
| name29 | 3 | 50 | 0 | 1 | G3P2 | 0 | 2 | 4 |
| name30 | 3 | 45 | 0 | 1 | G3P1 | 0 | 2 | 4 |
| name31 | 2 | 38 | 0 | 1 | G6P2 | 0 | 1 | 3 |
| name32 | 3 | 44 | 0 | 1 | G3P1 | 0 | 2 | 3 |
| name33 | 3 | 44 | 0 | 0 | G1P1 | 0 | 1 | 4 |
| name34 | 3 | 48 | 0 | 0 | G2P1 | 0 | 2 | 4 |
| name35 | 3 | 41 | 0 | 1 | G6P2 | 0 | 1 | 3 |
| name36 | 4 | 56 | 1 | 1 | G5P1 | 0 | 2 | 4 |
| name37 | 3 | 45 | 0 | 1 | G3P1 | 0 | 1 | 3 |
| name38 | 4 | 50 | 1 | 1 | G4P1 | 0 | 1 | 4 |
| name39 | 4 | 53 | 0 | 1 | G3P1 | 0 | 2 | 4 |
| name40 | 3 | 43 | 0 | 1 | G5P1 | 0 | 1 | 4 |
| name41 | 4 | 58 | 1 | 1 | G9P2 | 0 | 2 | 4 |
| name42 | 3 | 49 | 0 | 1 | G4P2 | 0 | 2 | 4 |
| name43 | 4 | 60 | 1 | 1 | G6P2 | 0 | 1 | 4 |
| name44 | 4 | 54 | 1 | 0 | G1P1 | 0 | 1 | 2 |
| name45 | 2 | 39 | 0 | 1 | G5P2 | 0 | 1 | 3 |
| name46 | 2 | 37 | 0 | 1 | G5P2 | 0 | 1 | 3 |
| name47 | 6 | 71 | 1 | 1 | G6P5 | 0 | 1 | 4 |
| name48 | 4 | 58 | 1 | 1 | G3P3 | 0 | 1 | 4 |
| name49 | 2 | 34 | 0 | 1 | G3P2 | 0 | 1 | 3 |
| name50 | 5 | 63 | 1 | 1 | G4P3 | 0 | 2 | 4 |
| name51 | 2 | 38 | 0 | 1 | G3P1 | 0 | 2 | 3 |
| name52 | 2 | 35 | 0 | 1 | G4P3 | 0 | 1 | 3 |
| name53 | 5 | 61 | 1 | 0 | G2P1 | 0 | 1 | 4 |
| name54 | 3 | 49 | 1 | 1 | G7P4 | 0 | 1 | 4 |
| name55 | 3 | 50 | 1 | 1 | G3P1 | 0 | 1 | 4 |
| name56 | 3 | 50 | 1 | 1 | G3P2 | 0 | 1 | 4 |
| name57 | 4 | 51 | 0 | 0 | G1P1 | 0 | 1 | 4 |
| name58 | 4 | 57 | 1 | 1 | G8P2 | 0 | 1 | 4 |
| name59 | 3 | 41 | 0 | 1 | G3P1 | 0 | 1 | 2 |
| name60 | 5 | 61 | 1 | 1 | G9P6 | 0 | 2 | 4 |
| name61 | 3 | 41 | 0 | 0 | G2P1 | 0 | 2 | 3 |
| name62 | 2 | 34 | 0 | 1 | G3P1 | 0 | 1 | 3 |
| name63 | 4 | 50 | 0 | 1 | G5P2 | 0 | 1 | 4 |
| name64 | 3 | 44 | 0 | 0 | G2P1 | 0 | 2 | 3 |
| name65 | 3 | 43 | 0 | 1 | G6P2 | 0 | 1 | 3 |
| name66 | 3 | 47 | 0 | 1 | G4P1 | 0 | 1 | 4 |
| name67 | 3 | 45 | 0 | 0 | G1P1 | 0 | 2 | 4 |
| name68 | 4 | 51 | 0 | 1 | G4P1 | 0 | 2 | 4 |
| name69 | 3 | 42 | 0 | 1 | G10P7 | 0 | 1 | 3 |
| name70 | 4 | 58 | 1 | 0 | G2P1 | 0 | 2 | 4 |
| name71 | 1 | 28 | 0 | 1 | G4P1 | 0 | 2 | 3 |
| name72 | 5 | 68 | 1 | 1 | G4P4 | 0 | 1 | 4 |
| name73 | 3 | 47 | 0 | 0 | G2P1 | 0 | 1 | 4 |
| name74 | 5 | 68 | 1 | 0 | G2P2 | 0 | 2 | 4 |
| name75 | 4 | 57 | 1 | 1 | G5P3 | 0 | 1 | 4 |
| name76 | 4 | 59 | 1 | 1 | G5P3 | 0 | 2 | 4 |
| name77 | 4 | 56 | 1 | 1 | G6P5 | 0 | 1 | 4 |
| name78 | 2 | 34 | 0 | 1 | G5P1 | 0 | 1 | 3 |
| name79 | 3 | 45 | 0 | 1 | G5P1 | 0 | 1 | 3 |
| name80 | 3 | 47 | 0 | 1 | G5P2 | 0 | 1 | 4 |
| name81 | 3 | 42 | 0 | 1 | G7P3 | 0 | 1 | 4 |
| name82 | 3 | 43 | 0 | 1 | G3P1 | 0 | 1 | 4 |
| name83 | 5 | 65 | 1 | 1 | G5P4 | 0 | 1 | 4 |
| name84 | 4 | 51 | 0 | 1 | G10P6 | 0 | 1 | 4 |
| name85 | 3 | 42 | 0 | 1 | G3P1 | 0 | 1 | 4 |
| name86 | 2 | 34 | 0 | 1 | G3P1 | 0 | 1 | 3 |
| name87 | 4 | 50 | 0 | 1 | G5P2 | 0 | 1 | 4 |
| name88 | 3 | 44 | 0 | 0 | G2P1 | 0 | 2 | 3 |
| name89 | 3 | 43 | 0 | 1 | G6P2 | 0 | 1 | 3 |
| name90 | 4 | 59 | 1 | 1 | G5P3 | 0 | 2 | 4 |
| name91 | 4 | 56 | 1 | 1 | G6P5 | 0 | 1 | 4 |
| name92 | 2 | 34 | 0 | 1 | G5P1 | 0 | 1 | 3 |
| name93 | 3 | 45 | 0 | 1 | G5P1 | 0 | 1 | 3 |
| name94 | 3 | 47 | 0 | 1 | G5P2 | 0 | 1 | 4 |
| name95 | 3 | 42 | 0 | 1 | G7P3 | 0 | 1 | 4 |
| name96 | 3 | 43 | 0 | 1 | G3P1 | 0 | 1 | 4 |
| name97 | 5 | 65 | 1 | 1 | G5P4 | 0 | 1 | 4 |
| name98 | 4 | 51 | 0 | 1 | G10P6 | 0 | 1 | 4 |
| name99 | 3 | 42 | 0 | 1 | G3P1 | 0 | 1 | 4 |
| name100 | 3 | 47 | 0 | 1 | G4P1 | 0 | 1 | 4 |
| name101 | 3 | 45 | 0 | 0 | G1P1 | 0 | 2 | 4 |
| name102 | 4 | 51 | 0 | 1 | G4P1 | 0 | 2 | 4 |
| name103 | 3 | 42 | 0 | 1 | G10P7 | 0 | 1 | 3 |
| name104 | 4 | 58 | 1 | 0 | G2P1 | 0 | 2 | 4 |
| name105 | 1 | 28 | 0 | 1 | G4P1 | 0 | 2 | 3 |
| name106 | 4 | 51 | 1 | 1 | G6P2 | 0 | 1 | 4 |
| name107 | 3 | 44 | 0 | 1 | G6P3 | 0 | 1 | 4 |
| name108 | 4 | 55 | 1 | 1 | G4P4 | 0 | 2 | 4 |
| name109 | 3 | 50 | 0 | 1 | G3P2 | 0 | 2 | 4 |
| name110 | 3 | 45 | 0 | 1 | G3P1 | 0 | 2 | 4 |
| name111 | 2 | 38 | 0 | 1 | G6P2 | 0 | 1 | 3 |
| name112 | 3 | 44 | 0 | 1 | G3P1 | 0 | 2 | 3 |
| name113 | 3 | 44 | 0 | 0 | G1P1 | 0 | 1 | 4 |
| name114 | 4 | 54 | 1 | 0 | G1P1 | 0 | 1 | 2 |
| name115 | 2 | 39 | 0 | 1 | G5P2 | 0 | 1 | 3 |
| name116 | 3 | 48 | 0 | 0 | G2P1 | 0 | 2 | 4 |
| name117 | 3 | 41 | 0 | 1 | G6P2 | 0 | 1 | 3 |
| name118 | 4 | 56 | 1 | 1 | G5P1 | 0 | 2 | 4 |
| name119 | 3 | 45 | 0 | 1 | G3P1 | 0 | 1 | 3 |
| name120 | 4 | 50 | 1 | 1 | G4P1 | 0 | 1 | 4 |
| name121 | 4 | 53 | 0 | 1 | G3P1 | 0 | 2 | 4 |
| name122 | 3 | 43 | 0 | 1 | G5P1 | 0 | 1 | 4 |
| name123 | 4 | 58 | 1 | 1 | G9P2 | 0 | 2 | 4 |
| name124 | 3 | 50 | 1 | 1 | G3P2 | 0 | 1 | 4 |
| name125 | 4 | 51 | 0 | 0 | G1P1 | 0 | 1 | 4 |
| name126 | 4 | 57 | 1 | 1 | G8P2 | 0 | 1 | 4 |
| name127 | 3 | 41 | 0 | 1 | G3P1 | 0 | 1 | 2 |
| name128 | 5 | 61 | 1 | 1 | G9P6 | 0 | 2 | 4 |
| name129 | 3 | 41 | 0 | 0 | G2P1 | 0 | 2 | 3 |
| name130 | 5 | 68 | 1 | 1 | G4P4 | 0 | 1 | 4 |
| name131 | 3 | 47 | 0 | 0 | G2P1 | 0 | 1 | 4 |
| name132 | 5 | 68 | 1 | 0 | G2P2 | 0 | 2 | 4 |
| name133 | 4 | 57 | 1 | 1 | G5P3 | 0 | 1 | 4 |
| name134 | 3 | 49 | 0 | 1 | G4P2 | 0 | 2 | 4 |
| name135 | 4 | 60 | 1 | 1 | G6P2 | 0 | 1 | 4 |
| name136 | 4 | 53 | 1 | 1 | G3P3 | 0 | 1 | 4 |
| name137 | 4 | 53 | 1 | 1 | G3P3 | 0 | 2 | 4 |
| name138 | 2 | 39 | 0 | 1 | G3P2 | 0 | 1 | 3 |
| name139 | 4 | 56 | 1 | 1 | G7P3 | 0 | 1 | 4 |
| name140 | 4 | 60 | 1 | 0 | G2P1 | 0 | 1 | 4 |
| name141 | 3 | 44 | 0 | 1 | G6P2 | 0 | 1 | 3 |
| name142 | 3 | 46 | 0 | 0 | G2P1 | 0 | 1 | 4 |
| name143 | 2 | 37 | 0 | 1 | G5P2 | 0 | 1 | 3 |
| name144 | 6 | 71 | 1 | 1 | G6P5 | 0 | 1 | 4 |
| name145 | 4 | 58 | 1 | 1 | G3P3 | 0 | 1 | 4 |
| name146 | 2 | 34 | 0 | 1 | G3P2 | 0 | 1 | 3 |
| name147 | 5 | 63 | 1 | 1 | G4P3 | 0 | 2 | 4 |
| name148 | 2 | 38 | 0 | 1 | G3P1 | 0 | 2 | 3 |
| name149 | 2 | 35 | 0 | 1 | G4P3 | 0 | 1 | 3 |
| name150 | 5 | 61 | 1 | 0 | G2P1 | 0 | 1 | 4 |
| name151 | 3 | 49 | 1 | 1 | G7P4 | 0 | 1 | 4 |
| name152 | 3 | 50 | 1 | 1 | G3P1 | 0 | 1 | 4 |
| name153 | 4 | 51 | 0 | 1 | G5P2 | 0 | 1 | 4 |
| name154 | 3 | 45 | 0 | 1 | G3P1 | 0 | 1 | 4 |
| name155 | 4 | 56 | 0 | 0 | G2P1 | 0 | 1 | 4 |
| name156 | 3 | 43 | 0 | 0 | G2P1 | 0 | 1 | 4 |
| name157 | 3 | 44 | 0 | 1 | G3P1 | 0 | 1 | 4 |
| name158 | 4 | 56 | 1 | 1 | G3P1 | 0 | 2 | 4 |
| name159 | 2 | 36 | 0 | 1 | G3P1 | 0 | 2 | 3 |
| name160 | 4 | 58 | 1 | 1 | G4P2 | 0 | 1 | 4 |
| name161 | 4 | 52 | 1 | 1 | G4P1 | 0 | 2 | 4 |
| name162 | 3 | 43 | 0 | 1 | G3P2 | 0 | 2 | 3 |
| name163 | 2 | 40 | 0 | 0 | G1P1 | 0 | 2 | 3 |
| name164 | 2 | 39 | 0 | 0 | G2P1 | 0 | 1 | 3 |
| name165 | 4 | 54 | 1 | 1 | G4P3 | 0 | 1 | 4 |
| name166 | 4 | 59 | 1 | 1 | G3P3 | 0 | 1 | 4 |
| name167 | 2 | 35 | 0 | 1 | G6P2 | 0 | 1 | 3 |
| name168 | 4 | 57 | 1 | 1 | G8P2 | 0 | 2 | 4 |
| name169 | 4 | 56 | 1 | 0 | G2P1 | 0 | 1 | 4 |
| name170 | 3 | 42 | 0 | 1 | G3P1 | 0 | 1 | 3 |

| 姓名 | 肿块大小 | 角化与否 | 病理类型 | 水平扩散 | 间质浸润深度 | 脉管浸润 | 淋巴转移 | 宫旁浸润 |
| --- | --- | --- | --- | --- | --- | --- | --- | --- |
| name | Tumor size | Keratosis | Pathologic type | Horizontal  diffusion | Depth of interstitial  infiltration | vascular  invasion | Lymph node  metastasis | Infiltration of  the uterus |
|  | ≤4cm=0 | No=0 | Squamous cell  carcinoma =1 | ≤4cm=1 | ≤5mm=1 | No=0 | No=0 | No=0 |
|  | ＞4cm=1 | Yes=1 |  | ＞4cm=2 | ＞5mm=2 | Yes=1 | Yes=1 | Yes=1 |
|  |  |  |  |  |  |  |  |  |
|  |  |  |  |  |  |  |  |  |
|  |  |  |  |  |  |  |  |  |
| name1 | 1 | 1 | 1 | 2 | 2 | 1 | 1 | 1 |
| name2 | 0 | 0 | 1 | 1 | 2 | 1 | 0 | 0 |
| name3 | 1 | 0 | 1 | 2 | 2 | 1 | 1 | 1 |
| name4 | 1 | 0 | 1 | 1 | 2 | 1 | 0 | 0 |
| name5 | 0 | 1 | 1 | 1 | 2 | 0 | 0 | 0 |
| name6 | 0 | 1 | 1 | 1 | 2 | 0 | 0 | 0 |
| name7 | 0 | 0 | 1 | 1 | 2 | 0 | 0 | 0 |
| name8 | 1 | 0 | 1 | 2 | 2 | 1 | 1 | 1 |
| name9 | 0 | 1 | 1 | 1 | 2 | 0 | 0 | 0 |
| name10 | 0 | 0 | 1 | 1 | 2 | 1 | 0 | 0 |
| name11 | 1 | 0 | 1 | 1 | 2 | 1 | 0 | 0 |
| name12 | 0 | 0 | 1 | 1 | 2 | 1 | 0 | 0 |
| name13 | 1 | 1 | 1 | 2 | 2 | 1 | 1 | 0 |
| name14 | 1 | 1 | 1 | 2 | 2 | 1 | 1 | 0 |
| name15 | 0 | 1 | 1 | 1 | 2 | 1 | 0 | 0 |
| name16 | 0 | 1 | 1 | 1 | 2 | 1 | 1 | 1 |
| name17 | 1 | 1 | 1 | 2 | 2 | 1 | 1 | 0 |
| name18 | 1 | 0 | 1 | 2 | 2 | 1 | 1 | 1 |
| name19 | 1 | 0 | 1 | 2 | 2 | 1 | 1 | 0 |
| name20 | 1 | 1 | 1 | 2 | 2 | 1 | 1 | 0 |
| name21 | 1 | 0 | 1 | 2 | 2 | 1 | 1 | 0 |
| name22 | 1 | 1 | 1 | 1 | 2 | 0 | 0 | 0 |
| name23 | 1 | 0 | 1 | 2 | 2 | 1 | 1 | 1 |
| name24 | 0 | 0 | 1 | 1 | 2 | 1 | 1 | 0 |
| name25 | 1 | 0 | 1 | 2 | 2 | 1 | 1 | 0 |
| name26 | 0 | 1 | 1 | 1 | 2 | 0 | 0 | 0 |
| name27 | 0 | 0 | 1 | 1 | 2 | 0 | 0 | 0 |
| name28 | 0 | 1 | 1 | 1 | 2 | 0 | 1 | 0 |
| name29 | 0 | 1 | 1 | 1 | 2 | 0 | 0 | 0 |
| name30 | 0 | 1 | 1 | 1 | 2 | 0 | 1 | 0 |
| name31 | 1 | 0 | 1 | 1 | 2 | 1 | 1 | 1 |
| name32 | 0 | 0 | 1 | 2 | 2 | 1 | 1 | 1 |
| name33 | 0 | 0 | 1 | 1 | 1 | 0 | 0 | 1 |
| name34 | 1 | 1 | 1 | 2 | 2 | 1 | 1 | 0 |
| name35 | 0 | 1 | 1 | 1 | 2 | 1 | 1 | 0 |
| name36 | 0 | 0 | 1 | 1 | 2 | 1 | 1 | 0 |
| name37 | 1 | 1 | 1 | 1 | 2 | 1 | 1 | 0 |
| name38 | 0 | 1 | 1 | 1 | 2 | 1 | 0 | 0 |
| name39 | 0 | 0 | 1 | 1 | 2 | 1 | 1 | 1 |
| name40 | 0 | 1 | 1 | 1 | 2 | 0 | 0 | 0 |
| name41 | 1 | 0 | 1 | 2 | 2 | 1 | 1 | 1 |
| name42 | 1 | 0 | 1 | 2 | 2 | 1 | 0 | 0 |
| name43 | 0 | 1 | 1 | 1 | 2 | 1 | 0 | 0 |
| name44 | 0 | 1 | 1 | 1 | 2 | 0 | 0 | 0 |
| name45 | 1 | 0 | 1 | 2 | 2 | 1 | 1 | 1 |
| name46 | 0 | 1 | 1 | 1 | 1 | 0 | 0 | 0 |
| name47 | 0 | 0 | 1 | 1 | 2 | 1 | 1 | 0 |
| name48 | 1 | 1 | 1 | 2 | 2 | 1 | 1 | 1 |
| name49 | 0 | 0 | 1 | 1 | 2 | 1 | 1 | 0 |
| name50 | 0 | 0 | 1 | 1 | 2 | 1 | 1 | 0 |
| name51 | 1 | 1 | 1 | 2 | 2 | 1 | 1 | 1 |
| name52 | 0 | 1 | 1 | 1 | 2 | 0 | 0 | 0 |
| name53 | 1 | 0 | 1 | 2 | 2 | 1 | 1 | 1 |
| name54 | 1 | 1 | 1 | 2 | 2 | 1 | 1 | 0 |
| name55 | 0 | 1 | 1 | 1 | 2 | 0 | 0 | 0 |
| name56 | 0 | 1 | 1 | 1 | 2 | 0 | 1 | 0 |
| name57 | 0 | 0 | 1 | 1 | 2 | 1 | 1 | 0 |
| name58 | 0 | 0 | 1 | 1 | 2 | 1 | 1 | 0 |
| name59 | 0 | 1 | 1 | 1 | 2 | 1 | 1 | 0 |
| name60 | 1 | 0 | 1 | 2 | 2 | 1 | 1 | 1 |
| name61 | 0 | 0 | 1 | 1 | 2 | 1 | 1 | 0 |
| name62 | 0 | 1 | 1 | 1 | 1 | 0 | 0 | 0 |
| name63 | 0 | 1 | 1 | 1 | 2 | 1 | 0 | 0 |
| name64 | 0 | 0 | 1 | 1 | 2 | 1 | 0 | 0 |
| name65 | 0 | 0 | 1 | 1 | 2 | 1 | 1 | 1 |
| name66 | 1 | 1 | 1 | 2 | 2 | 1 | 1 | 0 |
| name67 | 0 | 0 | 1 | 2 | 2 | 1 | 1 | 1 |
| name68 | 1 | 0 | 1 | 1 | 2 | 1 | 1 | 0 |
| name69 | 1 | 1 | 1 | 2 | 2 | 1 | 1 | 1 |
| name70 | 0 | 1 | 1 | 1 | 2 | 1 | 1 | 0 |
| name71 | 1 | 1 | 1 | 2 | 2 | 1 | 1 | 1 |
| name72 | 0 | 1 | 1 | 1 | 2 | 1 | 1 | 0 |
| name73 | 1 | 1 | 1 | 2 | 2 | 1 | 1 | 1 |
| name74 | 1 | 1 | 1 | 2 | 2 | 1 | 1 | 1 |
| name75 | 1 | 0 | 1 | 2 | 2 | 1 | 1 | 0 |
| name76 | 0 | 0 | 1 | 1 | 2 | 1 | 1 | 0 |
| name77 | 1 | 1 | 1 | 2 | 2 | 1 | 1 | 0 |
| name78 | 0 | 1 | 1 | 1 | 2 | 1 | 1 | 0 |
| name79 | 0 | 1 | 1 | 1 | 1 | 1 | 0 | 0 |
| name80 | 1 | 1 | 1 | 2 | 2 | 1 | 1 | 0 |
| name81 | 0 | 0 | 1 | 2 | 2 | 1 | 1 | 0 |
| name82 | 1 | 1 | 1 | 2 | 2 | 1 | 1 | 0 |
| name83 | 0 | 0 | 1 | 1 | 2 | 1 | 1 | 0 |
| name84 | 0 | 0 | 1 | 1 | 2 | 1 | 1 | 0 |
| name85 | 1 | 1 | 1 | 2 | 2 | 1 | 1 | 0 |
| name86 | 0 | 1 | 1 | 1 | 1 | 0 | 0 | 0 |
| name87 | 0 | 1 | 1 | 1 | 2 | 1 | 0 | 0 |
| name88 | 0 | 0 | 1 | 1 | 2 | 1 | 0 | 0 |
| name89 | 0 | 0 | 1 | 1 | 2 | 1 | 1 | 1 |
| name90 | 0 | 0 | 1 | 1 | 2 | 1 | 1 | 0 |
| name91 | 1 | 1 | 1 | 2 | 2 | 1 | 1 | 0 |
| name92 | 0 | 1 | 1 | 1 | 2 | 1 | 1 | 0 |
| name93 | 0 | 1 | 1 | 1 | 1 | 1 | 0 | 0 |
| name94 | 1 | 1 | 1 | 2 | 2 | 1 | 1 | 0 |
| name95 | 0 | 0 | 1 | 2 | 2 | 1 | 1 | 0 |
| name96 | 1 | 1 | 1 | 2 | 2 | 1 | 1 | 0 |
| name97 | 0 | 0 | 1 | 1 | 2 | 1 | 1 | 0 |
| name98 | 0 | 0 | 1 | 1 | 2 | 1 | 1 | 0 |
| name99 | 1 | 1 | 1 | 2 | 2 | 1 | 1 | 0 |
| name100 | 1 | 1 | 1 | 2 | 2 | 1 | 1 | 0 |
| name101 | 0 | 0 | 1 | 2 | 2 | 1 | 1 | 1 |
| name102 | 1 | 0 | 1 | 1 | 2 | 1 | 1 | 0 |
| name103 | 1 | 1 | 1 | 2 | 2 | 1 | 1 | 1 |
| name104 | 0 | 1 | 1 | 1 | 2 | 1 | 1 | 0 |
| name105 | 1 | 1 | 1 | 2 | 2 | 1 | 1 | 1 |
| name106 | 0 | 1 | 1 | 1 | 2 | 0 | 0 | 0 |
| name107 | 0 | 0 | 1 | 1 | 2 | 0 | 0 | 0 |
| name108 | 0 | 1 | 1 | 1 | 2 | 0 | 1 | 0 |
| name109 | 0 | 1 | 1 | 1 | 2 | 0 | 0 | 0 |
| name110 | 0 | 1 | 1 | 1 | 2 | 0 | 1 | 0 |
| name111 | 1 | 0 | 1 | 1 | 2 | 1 | 1 | 1 |
| name112 | 0 | 0 | 1 | 2 | 2 | 1 | 1 | 1 |
| name113 | 0 | 0 | 1 | 1 | 1 | 0 | 0 | 1 |
| name114 | 0 | 1 | 1 | 1 | 2 | 0 | 0 | 0 |
| name115 | 1 | 0 | 1 | 2 | 2 | 1 | 1 | 1 |
| name116 | 1 | 1 | 1 | 2 | 2 | 1 | 1 | 0 |
| name117 | 0 | 1 | 1 | 1 | 2 | 1 | 1 | 0 |
| name118 | 0 | 0 | 1 | 1 | 2 | 1 | 1 | 0 |
| name119 | 1 | 1 | 1 | 1 | 2 | 1 | 1 | 0 |
| name120 | 0 | 1 | 1 | 1 | 2 | 1 | 0 | 0 |
| name121 | 0 | 0 | 1 | 1 | 2 | 1 | 1 | 1 |
| name122 | 0 | 1 | 1 | 1 | 2 | 0 | 0 | 0 |
| name123 | 1 | 0 | 1 | 2 | 2 | 1 | 1 | 1 |
| name124 | 0 | 1 | 1 | 1 | 2 | 0 | 1 | 0 |
| name125 | 0 | 0 | 1 | 1 | 2 | 1 | 1 | 0 |
| name126 | 0 | 0 | 1 | 1 | 2 | 1 | 1 | 0 |
| name127 | 0 | 1 | 1 | 1 | 2 | 1 | 1 | 0 |
| name128 | 1 | 0 | 1 | 2 | 2 | 1 | 1 | 1 |
| name129 | 0 | 0 | 1 | 1 | 2 | 1 | 1 | 0 |
| name130 | 0 | 1 | 1 | 1 | 2 | 1 | 1 | 0 |
| name131 | 1 | 1 | 1 | 2 | 2 | 1 | 1 | 1 |
| name132 | 1 | 1 | 1 | 2 | 2 | 1 | 1 | 1 |
| name133 | 1 | 0 | 1 | 2 | 2 | 1 | 1 | 0 |
| name134 | 1 | 0 | 1 | 2 | 2 | 1 | 0 | 0 |
| name135 | 0 | 1 | 1 | 1 | 2 | 1 | 0 | 0 |
| name136 | 0 | 0 | 1 | 1 | 2 | 1 | 0 | 0 |
| name137 | 1 | 0 | 1 | 2 | 2 | 1 | 1 | 1 |
| name138 | 1 | 0 | 1 | 1 | 2 | 1 | 0 | 0 |
| name139 | 0 | 1 | 1 | 1 | 2 | 0 | 0 | 0 |
| name140 | 0 | 1 | 1 | 1 | 2 | 0 | 0 | 0 |
| name141 | 0 | 0 | 1 | 1 | 2 | 0 | 0 | 0 |
| name142 | 1 | 0 | 1 | 2 | 2 | 1 | 1 | 1 |
| name143 | 0 | 1 | 1 | 1 | 1 | 0 | 0 | 0 |
| name144 | 0 | 0 | 1 | 1 | 2 | 1 | 1 | 0 |
| name145 | 1 | 1 | 1 | 2 | 2 | 1 | 1 | 1 |
| name146 | 0 | 0 | 1 | 1 | 2 | 1 | 1 | 0 |
| name147 | 0 | 0 | 1 | 1 | 2 | 1 | 1 | 0 |
| name148 | 1 | 1 | 1 | 2 | 2 | 1 | 1 | 1 |
| name149 | 0 | 1 | 1 | 1 | 2 | 0 | 0 | 0 |
| name150 | 1 | 0 | 1 | 2 | 2 | 1 | 1 | 1 |
| name151 | 1 | 1 | 1 | 2 | 2 | 1 | 1 | 0 |
| name152 | 0 | 1 | 1 | 1 | 2 | 0 | 0 | 0 |
| name153 | 0 | 1 | 1 | 1 | 2 | 0 | 0 | 0 |
| name154 | 0 | 0 | 1 | 1 | 2 | 1 | 0 | 0 |
| name155 | 1 | 0 | 1 | 1 | 2 | 1 | 0 | 0 |
| name156 | 0 | 0 | 1 | 1 | 2 | 1 | 0 | 0 |
| name157 | 1 | 1 | 1 | 2 | 2 | 1 | 1 | 0 |
| name158 | 1 | 1 | 1 | 2 | 2 | 1 | 1 | 0 |
| name159 | 0 | 1 | 1 | 1 | 2 | 1 | 0 | 0 |
| name160 | 0 | 1 | 1 | 1 | 2 | 1 | 1 | 1 |
| name161 | 1 | 1 | 1 | 2 | 2 | 1 | 1 | 0 |
| name162 | 1 | 1 | 1 | 2 | 2 | 1 | 1 | 1 |
| name163 | 1 | 0 | 1 | 2 | 2 | 1 | 1 | 1 |
| name164 | 1 | 0 | 1 | 2 | 2 | 1 | 1 | 0 |
| name165 | 1 | 1 | 1 | 2 | 2 | 1 | 1 | 0 |
| name166 | 1 | 0 | 1 | 2 | 2 | 1 | 1 | 0 |
| name167 | 1 | 1 | 1 | 1 | 2 | 0 | 0 | 0 |
| name168 | 1 | 0 | 1 | 2 | 2 | 1 | 1 | 1 |
| name169 | 0 | 0 | 1 | 1 | 2 | 1 | 1 | 0 |
| name170 | 1 | 0 | 1 | 2 | 2 | 1 | 1 | 0 |

| 姓名 | 宫旁切缘 | 阴道累及 | 阴道切缘 | 组织学分级 | HPV | 术后放疗 | 术后化疗 |
| --- | --- | --- | --- | --- | --- | --- | --- |
| name | Margin of  uterus | Vaginal  involvement | Vagina  cutting  edge | Histological  grading | HPV | Postoperative  radiotherapy | Postoperative  chemotherapy |
|  | Negative=0 | No=0 | Negative=0 | High=1 | Negative=0 | No=0 | No=0 |
|  | Positive=1 | Yes=1 | Positive=1 | Medium=2 | Positive=1 | Yes=1 | Yes=1 |
|  |  |  |  | Low=3 |  |  |  |
|  |  |  |  |  |  |  |  |
|  |  |  |  |  |  |  |  |
| name1 | 0 | 1 | 0 | 2 | 1 | 1 | 1 |
| name2 | 0 | 0 | 1 | 3 | 1 | 0 | 0 |
| name3 | 0 | 1 | 1 | 2 | 1 | 0 | 0 |
| name4 | 0 | 0 | 0 | 3 | 1 | 0 | 0 |
| name5 | 0 | 0 | 0 | 1 | 1 | 0 | 0 |
| name6 | 0 | 0 | 0 | 1 | 1 | 1 | 1 |
| name7 | 0 | 0 | 0 | 3 | 1 | 1 | 1 |
| name8 | 0 | 1 | 0 | 3 | 1 | 1 | 1 |
| name9 | 0 | 0 | 0 | 3 | 1 | 0 | 0 |
| name10 | 0 | 0 | 0 | 3 | 1 | 1 | 1 |
| name11 | 0 | 0 | 0 | 3 | 1 | 0 | 0 |
| name12 | 0 | 0 | 0 | 3 | 1 | 0 | 0 |
| name13 | 0 | 1 | 0 | 2 | 1 | 0 | 0 |
| name14 | 0 | 1 | 0 | 2 | 1 | 0 | 0 |
| name15 | 0 | 0 | 0 | 2 | 1 | 1 | 1 |
| name16 | 0 | 0 | 0 | 2 | 1 | 0 | 0 |
| name17 | 0 | 1 | 0 | 2 | 1 | 0 | 0 |
| name18 | 1 | 1 | 0 | 2 | 1 | 0 | 0 |
| name19 | 0 | 1 | 1 | 2 | 1 | 1 | 1 |
| name20 | 0 | 1 | 0 | 3 | 1 | 0 | 0 |
| name21 | 0 | 1 | 0 | 3 | 1 | 0 | 0 |
| name22 | 0 | 0 | 0 | 2 | 1 | 0 | 0 |
| name23 | 1 | 1 | 0 | 3 | 1 | 0 | 0 |
| name24 | 0 | 1 | 1 | 3 | 1 | 0 | 0 |
| name25 | 0 | 1 | 0 | 2 | 1 | 0 | 0 |
| name26 | 0 | 0 | 0 | 1 | 1 | 0 | 0 |
| name27 | 0 | 0 | 0 | 2 | 1 | 0 | 1 |
| name28 | 0 | 1 | 1 | 2 | 1 | 0 | 1 |
| name29 | 0 | 0 | 0 | 1 | 1 | 0 | 1 |
| name30 | 0 | 0 | 0 | 3 | 1 | 1 | 1 |
| name31 | 1 | 1 | 1 | 3 | 1 | 0 | 0 |
| name32 | 1 | 0 | 0 | 3 | 1 | 0 | 1 |
| name33 | 0 | 0 | 0 | 3 | 1 | 0 | 1 |
| name34 | 0 | 1 | 0 | 2 | 1 | 1 | 1 |
| name35 | 0 | 0 | 0 | 2 | 1 | 1 | 1 |
| name36 | 0 | 0 | 0 | 3 | 1 | 0 | 0 |
| name37 | 0 | 0 | 0 | 3 | 1 | 1 | 1 |
| name38 | 0 | 0 | 0 | 1 | 1 | 0 | 0 |
| name39 | 0 | 0 | 0 | 3 | 1 | 0 | 0 |
| name40 | 0 | 0 | 0 | 2 | 0 | 1 | 1 |
| name41 | 0 | 1 | 0 | 3 | 0 | 1 | 1 |
| name42 | 0 | 1 | 0 | 3 | 0 | 1 | 1 |
| name43 | 0 | 1 | 0 | 2 | 0 | 1 | 1 |
| name44 | 0 | 0 | 0 | 3 | 0 | 0 | 0 |
| name45 | 1 | 1 | 1 | 1 | 1 | 1 | 1 |
| name46 | 0 | 0 | 0 | 2 | 1 | 0 | 0 |
| name47 | 0 | 1 | 0 | 3 | 1 | 1 | 1 |
| name48 | 0 | 1 | 0 | 2 | 1 | 1 | 1 |
| name49 | 0 | 0 | 0 | 3 | 1 | 1 | 1 |
| name50 | 0 | 1 | 0 | 3 | 1 | 1 | 1 |
| name51 | 1 | 1 | 1 | 3 | 1 | 0 | 0 |
| name52 | 0 | 0 | 0 | 1 | 1 | 1 | 1 |
| name53 | 0 | 1 | 0 | 2 | 1 | 1 | 1 |
| name54 | 0 | 1 | 0 | 3 | 1 | 0 | 0 |
| name55 | 0 | 0 | 0 | 1 | 1 | 1 | 1 |
| name56 | 0 | 0 | 0 | 2 | 1 | 0 | 0 |
| name57 | 0 | 1 | 0 | 3 | 1 | 1 | 1 |
| name58 | 0 | 0 | 1 | 3 | 1 | 1 | 1 |
| name59 | 0 | 0 | 0 | 1 | 1 | 1 | 0 |
| name60 | 0 | 1 | 0 | 3 | 1 | 0 | 0 |
| name61 | 0 | 0 | 0 | 3 | 1 | 0 | 1 |
| name62 | 0 | 0 | 0 | 1 | 1 | 0 | 0 |
| name63 | 0 | 0 | 0 | 1 | 0 | 1 | 0 |
| name64 | 0 | 0 | 0 | 3 | 1 | 1 | 1 |
| name65 | 1 | 1 | 1 | 3 | 1 | 1 | 1 |
| name66 | 0 | 1 | 0 | 3 | 0 | 1 | 1 |
| name67 | 0 | 1 | 0 | 3 | 1 | 1 | 1 |
| name68 | 0 | 0 | 0 | 3 | 1 | 0 | 0 |
| name69 | 0 | 1 | 0 | 3 | 1 | 1 | 1 |
| name70 | 0 | 1 | 1 | 3 | 0 | 0 | 0 |
| name71 | 1 | 1 | 1 | 3 | 1 | 1 | 1 |
| name72 | 0 | 0 | 0 | 1 | 1 | 1 | 1 |
| name73 | 1 | 1 | 0 | 2 | 1 | 0 | 0 |
| name74 | 0 | 1 | 0 | 3 | 1 | 1 | 1 |
| name75 | 0 | 1 | 0 | 3 | 1 | 0 | 0 |
| name76 | 0 | 1 | 0 | 3 | 1 | 1 | 1 |
| name77 | 0 | 1 | 0 | 1 | 1 | 1 | 1 |
| name78 | 0 | 0 | 0 | 2 | 1 | 0 | 0 |
| name79 | 0 | 0 | 0 | 1 | 1 | 0 | 0 |
| name80 | 0 | 1 | 0 | 1 | 1 | 0 | 0 |
| name81 | 0 | 1 | 0 | 3 | 1 | 1 | 0 |
| name82 | 0 | 1 | 0 | 2 | 1 | 1 | 1 |
| name83 | 0 | 1 | 1 | 2 | 1 | 1 | 1 |
| name84 | 0 | 1 | 0 | 3 | 1 | 1 | 1 |
| name85 | 0 | 1 | 0 | 2 | 1 | 1 | 1 |
| name86 | 0 | 0 | 0 | 1 | 1 | 0 | 0 |
| name87 | 0 | 0 | 0 | 1 | 1 | 1 | 0 |
| name88 | 0 | 0 | 0 | 3 | 1 | 1 | 1 |
| name89 | 1 | 1 | 1 | 3 | 1 | 1 | 1 |
| name90 | 0 | 1 | 0 | 3 | 1 | 1 | 1 |
| name91 | 0 | 1 | 0 | 1 | 1 | 1 | 1 |
| name92 | 0 | 0 | 0 | 2 | 1 | 0 | 0 |
| name93 | 0 | 0 | 0 | 1 | 1 | 0 | 0 |
| name94 | 0 | 1 | 0 | 1 | 1 | 0 | 0 |
| name95 | 0 | 1 | 0 | 3 | 1 | 1 | 0 |
| name96 | 0 | 1 | 0 | 2 | 1 | 1 | 1 |
| name97 | 0 | 1 | 1 | 2 | 1 | 1 | 1 |
| name98 | 0 | 1 | 0 | 3 | 1 | 1 | 1 |
| name99 | 0 | 1 | 0 | 2 | 1 | 1 | 1 |
| name100 | 0 | 1 | 0 | 3 | 1 | 1 | 1 |
| name101 | 0 | 1 | 0 | 3 | 1 | 1 | 1 |
| name102 | 0 | 0 | 0 | 3 | 1 | 0 | 0 |
| name103 | 0 | 1 | 0 | 3 | 1 | 1 | 1 |
| name104 | 0 | 1 | 1 | 3 | 1 | 0 | 0 |
| name105 | 1 | 1 | 1 | 3 | 1 | 1 | 1 |
| name106 | 0 | 0 | 0 | 1 | 1 | 0 | 0 |
| name107 | 0 | 0 | 0 | 2 | 1 | 0 | 1 |
| name108 | 0 | 1 | 1 | 2 | 1 | 0 | 1 |
| name109 | 0 | 0 | 0 | 1 | 1 | 0 | 1 |
| name110 | 0 | 0 | 0 | 3 | 1 | 1 | 1 |
| name111 | 1 | 1 | 1 | 3 | 1 | 0 | 0 |
| name112 | 1 | 0 | 0 | 3 | 1 | 0 | 1 |
| name113 | 0 | 0 | 0 | 3 | 1 | 0 | 1 |
| name114 | 0 | 0 | 0 | 3 | 1 | 0 | 0 |
| name115 | 1 | 1 | 1 | 1 | 1 | 1 | 1 |
| name116 | 0 | 1 | 0 | 2 | 1 | 1 | 1 |
| name117 | 0 | 0 | 0 | 2 | 1 | 1 | 1 |
| name118 | 0 | 0 | 0 | 3 | 1 | 0 | 0 |
| name119 | 0 | 0 | 0 | 3 | 1 | 1 | 1 |
| name120 | 0 | 0 | 0 | 1 | 1 | 0 | 0 |
| name121 | 0 | 0 | 0 | 3 | 1 | 0 | 0 |
| name122 | 0 | 0 | 0 | 2 | 1 | 1 | 1 |
| name123 | 0 | 1 | 0 | 3 | 1 | 1 | 1 |
| name124 | 0 | 0 | 0 | 2 | 1 | 0 | 0 |
| name125 | 0 | 1 | 0 | 3 | 0 | 1 | 1 |
| name126 | 0 | 0 | 1 | 3 | 0 | 1 | 1 |
| name127 | 0 | 0 | 0 | 1 | 0 | 1 | 0 |
| name128 | 0 | 1 | 0 | 3 | 0 | 0 | 0 |
| name129 | 0 | 0 | 0 | 3 | 0 | 0 | 1 |
| name130 | 0 | 0 | 0 | 1 | 1 | 1 | 1 |
| name131 | 1 | 1 | 0 | 2 | 1 | 0 | 0 |
| name132 | 0 | 1 | 0 | 3 | 1 | 1 | 1 |
| name133 | 0 | 1 | 0 | 3 | 1 | 0 | 0 |
| name134 | 0 | 1 | 0 | 3 | 1 | 1 | 1 |
| name135 | 0 | 1 | 0 | 2 | 1 | 1 | 1 |
| name136 | 0 | 0 | 1 | 3 | 1 | 0 | 0 |
| name137 | 0 | 1 | 1 | 2 | 1 | 0 | 0 |
| name138 | 0 | 0 | 0 | 3 | 1 | 0 | 0 |
| name139 | 0 | 0 | 0 | 1 | 1 | 0 | 0 |
| name140 | 0 | 0 | 0 | 1 | 1 | 1 | 1 |
| name141 | 0 | 0 | 0 | 3 | 1 | 1 | 1 |
| name142 | 0 | 1 | 0 | 3 | 1 | 1 | 1 |
| name143 | 0 | 0 | 0 | 2 | 1 | 0 | 0 |
| name144 | 0 | 1 | 0 | 3 | 1 | 1 | 1 |
| name145 | 0 | 1 | 0 | 2 | 1 | 1 | 1 |
| name146 | 0 | 0 | 0 | 3 | 1 | 1 | 1 |
| name147 | 0 | 1 | 0 | 3 | 1 | 1 | 1 |
| name148 | 1 | 1 | 1 | 3 | 0 | 0 | 0 |
| name149 | 0 | 0 | 0 | 1 | 1 | 1 | 1 |
| name150 | 0 | 1 | 0 | 2 | 1 | 1 | 1 |
| name151 | 0 | 1 | 0 | 3 | 0 | 0 | 0 |
| name152 | 0 | 0 | 0 | 1 | 1 | 1 | 1 |
| name153 | 0 | 0 | 0 | 3 | 1 | 0 | 0 |
| name154 | 0 | 0 | 0 | 3 | 1 | 1 | 1 |
| name155 | 0 | 0 | 0 | 3 | 0 | 0 | 0 |
| name156 | 0 | 0 | 0 | 3 | 1 | 0 | 0 |
| name157 | 0 | 1 | 0 | 2 | 1 | 0 | 0 |
| name158 | 0 | 1 | 0 | 2 | 1 | 0 | 0 |
| name159 | 0 | 0 | 0 | 2 | 1 | 1 | 1 |
| name160 | 0 | 0 | 0 | 2 | 1 | 0 | 0 |
| name161 | 0 | 1 | 0 | 2 | 1 | 0 | 0 |
| name162 | 0 | 1 | 0 | 2 | 1 | 1 | 1 |
| name163 | 1 | 1 | 0 | 2 | 1 | 0 | 0 |
| name164 | 0 | 1 | 1 | 2 | 1 | 1 | 1 |
| name165 | 0 | 1 | 0 | 3 | 1 | 0 | 0 |
| name166 | 0 | 1 | 0 | 3 | 1 | 0 | 0 |
| name167 | 0 | 0 | 0 | 2 | 1 | 0 | 0 |
| name168 | 1 | 1 | 0 | 3 | 1 | 0 | 0 |
| name169 | 0 | 1 | 1 | 3 | 1 | 0 | 0 |
| name170 | 0 | 1 | 0 | 2 | 1 | 0 | 0 |
|  |  |  |  |  |  |  |  |

| 姓名 | 着色强度 | 病理诊断（金标准） | 免疫诊断分组 | 术后随访时间(月) | 预后 |
| --- | --- | --- | --- | --- | --- |
| name | Coloring | Pathological  diagnosis  (gold standard) | Immune  diagnostic  grouping | Postoperative  follow-up time  (month) | prognosis |
|  | Negative=0 | Squamous cell  carcinoma =1 | Negative=0 |  | No death =0 |
|  | Low positive=1 |  | Positive=1 |  | Died =1 |
|  | Medium positive=2 |  |  |  | Lost =2 |
|  | High positive=3 |  |  |  | Recurrence=3 |
|  |  |  |  |  |  |
| name1 | 2 | 1 | 1 | 39 | 0 |
| name2 | 0 | 1 | 1 | 38 | 0 |
| name3 | 2 | 1 | 1 | 30 | 2 |
| name4 | 3 | 1 | 1 | 21 | 3 |
| name5 | 2 | 1 | 1 | 11 | 2 |
| name6 | 3 | 1 | 0 | 25 | 2 |
| name7 | 0 | 1 | 1 | 21 | 3 |
| name8 | 3 | 1 | 1 | 39 | 0 |
| name9 | 3 | 1 | 1 | 21 | 3 |
| name10 | 2 | 1 | 0 | 38 | 0 |
| name11 | 2 | 1 | 1 | 39 | 0 |
| name12 | 2 | 1 | 0 | 12 | 2 |
| name13 | 2 | 1 | 1 | 40 | 0 |
| name14 | 3 | 1 | 1 | 42 | 0 |
| name15 | 3 | 1 | 1 | 44 | 0 |
| name16 | 2 | 1 | 0 | 46 | 0 |
| name17 | 3 | 1 | 1 | 21 | 3 |
| name18 | 3 | 1 | 0 | 47 | 0 |
| name19 | 3 | 1 | 0 | 23 | 2 |
| name20 | 3 | 1 | 0 | 40 | 0 |
| name21 | 3 | 1 | 0 | 39 | 0 |
| name22 | 0 | 1 | 1 | 38 | 0 |
| name23 | 3 | 1 | 0 | 38 | 0 |
| name24 | 2 | 1 | 1 | 39 | 0 |
| name25 | 3 | 1 | 1 | 37 | 0 |
| name26 | 0 | 1 | 1 | 36 | 0 |
| name27 | 3 | 1 | 1 | 38 | 0 |
| name28 | 0 | 1 | 1 | 37 | 0 |
| name29 | 2 | 1 | 1 | 15 | 2 |
| name30 | 2 | 1 | 1 | 40 | 0 |
| name31 | 3 | 1 | 1 | 38 | 0 |
| name32 | 0 | 1 | 0 | 35 | 0 |
| name33 | 2 | 1 | 0 | 46 | 0 |
| name34 | 3 | 1 | 1 | 45 | 0 |
| name35 | 2 | 1 | 0 | 24 | 2 |
| name36 | 0 | 1 | 1 | 45 | 0 |
| name37 | 2 | 1 | 1 | 44 | 0 |
| name38 | 0 | 1 | 1 | 42 | 0 |
| name39 | 3 | 1 | 1 | 9 | 1 |
| name40 | 2 | 1 | 1 | 18 | 3 |
| name41 | 2 | 1 | 0 | 38 | 0 |
| name42 | 2 | 1 | 0 | 37 | 0 |
| name43 | 2 | 1 | 1 | 21 | 2 |
| name44 | 0 | 1 | 1 | 19 | 3 |
| name45 | 0 | 1 | 0 | 18 | 2 |
| name46 | 0 | 1 | 0 | 39 | 0 |
| name47 | 0 | 1 | 0 | 46 | 0 |
| name48 | 2 | 1 | 1 | 10 | 1 |
| name49 | 0 | 1 | 0 | 43 | 0 |
| name50 | 2 | 1 | 1 | 40 | 0 |
| name51 | 3 | 1 | 1 | 7 | 1 |
| name52 | 3 | 1 | 1 | 21 | 3 |
| name53 | 2 | 1 | 1 | 35 | 0 |
| name54 | 3 | 1 | 1 | 37 | 0 |
| name55 | 2 | 1 | 1 | 16 | 2 |
| name56 | 2 | 1 | 1 | 37 | 0 |
| name57 | 2 | 1 | 1 | 1 | 1 |
| name58 | 0 | 1 | 1 | 36 | 0 |
| name59 | 2 | 1 | 1 | 36 | 0 |
| name60 | 3 | 1 | 1 | 1 | 1 |
| name61 | 0 | 1 | 0 | 36 | 0 |
| name62 | 2 | 1 | 1 | 28 | 3 |
| name63 | 3 | 1 | 1 | 13 | 3 |
| name64 | 2 | 1 | 1 | 37 | 0 |
| name65 | 3 | 1 | 1 | 38 | 0 |
| name66 | 3 | 1 | 1 | 36 | 0 |
| name67 | 2 | 1 | 1 | 22 | 3 |
| name68 | 2 | 1 | 1 | 17 | 3 |
| name69 | 3 | 1 | 1 | 37 | 0 |
| name70 | 3 | 1 | 0 | 25 | 2 |
| name71 | 3 | 1 | 1 | 25 | 3 |
| name72 | 3 | 1 | 1 | 11 | 3 |
| name73 | 3 | 1 | 1 | 22 | 3 |
| name74 | 3 | 1 | 0 | 37 | 0 |
| name75 | 3 | 1 | 1 | 9 | 3 |
| name76 | 2 | 1 | 0 | 39 | 0 |
| name77 | 3 | 1 | 1 | 37 | 0 |
| name78 | 3 | 1 | 1 | 10 | 1 |
| name79 | 2 | 1 | 1 | 11 | 1 |
| name80 | 3 | 1 | 0 | 36 | 0 |
| name81 | 2 | 1 | 1 | 38 | 0 |
| name82 | 3 | 1 | 0 | 42 | 0 |
| name83 | 2 | 1 | 0 | 22 | 2 |
| name84 | 0 | 1 | 0 | 41 | 0 |
| name85 | 3 | 1 | 0 | 40 | 0 |
| name86 | 2 | 1 | 1 | 11 | 1 |
| name87 | 3 | 1 | 0 | 40 | 0 |
| name88 | 2 | 1 | 1 | 41 | 0 |
| name89 | 3 | 1 | 1 | 8 | 1 |
| name90 | 2 | 1 | 1 | 39 | 0 |
| name91 | 3 | 1 | 1 | 37 | 0 |
| name92 | 3 | 1 | 1 | 38 | 0 |
| name93 | 2 | 1 | 1 | 36 | 0 |
| name94 | 3 | 1 | 1 | 38 | 0 |
| name95 | 2 | 1 | 1 | 35 | 0 |
| name96 | 3 | 1 | 0 | 36 | 0 |
| name97 | 2 | 1 | 0 | 39 | 0 |
| name98 | 0 | 1 | 1 | 37 | 0 |
| name99 | 3 | 1 | 0 | 22 | 2 |
| name100 | 3 | 1 | 1 | 33 | 0 |
| name101 | 2 | 1 | 1 | 42 | 0 |
| name102 | 2 | 1 | 1 | 13 | 1 |
| name103 | 3 | 1 | 1 | 11 | 1 |
| name104 | 3 | 1 | 1 | 34 | 0 |
| name105 | 3 | 1 | 0 | 35 | 0 |
| name106 | 0 | 1 | 0 | 36 | 0 |
| name107 | 3 | 1 | 1 | 19 | 2 |
| name108 | 0 | 1 | 1 | 39 | 0 |
| name109 | 2 | 1 | 0 | 21 | 1 |
| name110 | 2 | 1 | 0 | 40 | 0 |
| name111 | 3 | 1 | 0 | 40 | 0 |
| name112 | 0 | 1 | 1 | 9 | 1 |
| name113 | 2 | 1 | 0 | 41 | 0 |
| name114 | 0 | 1 | 1 | 41 | 0 |
| name115 | 0 | 1 | 1 | 10 | 1 |
| name116 | 3 | 1 | 1 | 9 | 1 |
| name117 | 2 | 1 | 1 | 44 | 0 |
| name118 | 0 | 1 | 1 | 20 | 3 |
| name119 | 2 | 1 | 1 | 36 | 2 |
| name120 | 0 | 1 | 1 | 39 | 0 |
| name121 | 3 | 1 | 1 | 9 | 1 |
| name122 | 2 | 1 | 1 | 10 | 1 |
| name123 | 2 | 1 | 1 | 9 | 1 |
| name124 | 2 | 1 | 1 | 34 | 0 |
| name125 | 2 | 1 | 0 | 24 | 1 |
| name126 | 0 | 1 | 1 | 12 | 1 |
| name127 | 2 | 1 | 1 | 25 | 3 |
| name128 | 3 | 1 | 1 | 10 | 1 |
| name129 | 0 | 1 | 1 | 37 | 0 |
| name130 | 3 | 1 | 1 | 39 | 0 |
| name131 | 3 | 1 | 1 | 30 | 2 |
| name132 | 3 | 1 | 1 | 44 | 0 |
| name133 | 3 | 1 | 1 | 9 | 1 |
| name134 | 2 | 1 | 0 | 42 | 0 |
| name135 | 2 | 1 | 1 | 25 | 2 |
| name136 | 0 | 1 | 1 | 44 | 0 |
| name137 | 2 | 1 | 1 | 43 | 0 |
| name138 | 3 | 1 | 0 | 43 | 0 |
| name139 | 2 | 1 | 1 | 40 | 0 |
| name140 | 3 | 1 | 0 | 22 | 1 |
| name141 | 0 | 1 | 1 | 44 | 0 |
| name142 | 3 | 1 | 1 | 44 | 0 |
| name143 | 0 | 1 | 1 | 10 | 1 |
| name144 | 0 | 1 | 0 | 24 | 0 |
| name145 | 2 | 1 | 1 | 10 | 1 |
| name146 | 0 | 1 | 0 | 36 | 0 |
| name147 | 2 | 1 | 0 | 30 | 2 |
| name148 | 3 | 1 | 0 | 38 | 0 |
| name149 | 3 | 1 | 0 | 38 | 0 |
| name150 | 2 | 1 | 1 | 36 | 0 |
| name151 | 3 | 1 | 0 | 37 | 0 |
| name152 | 2 | 1 | 1 | 35 | 0 |
| name153 | 3 | 1 | 1 | 15 | 2 |
| name154 | 2 | 1 | 1 | 42 | 0 |
| name155 | 2 | 1 | 1 | 41 | 0 |
| name156 | 2 | 1 | 1 | 40 | 0 |
| name157 | 2 | 1 | 1 | 40 | 0 |
| name158 | 3 | 1 | 1 | 43 | 0 |
| name159 | 3 | 1 | 1 | 22 | 3 |
| name160 | 2 | 1 | 0 | 41 | 0 |
| name161 | 3 | 1 | 0 | 40 | 0 |
| name162 | 2 | 1 | 1 | 36 | 0 |
| name163 | 3 | 1 | 0 | 15 | 2 |
| name164 | 3 | 1 | 1 | 36 | 0 |
| name165 | 3 | 1 | 1 | 37 | 0 |
| name166 | 3 | 1 | 1 | 35 | 0 |
| name167 | 0 | 1 | 1 | 36 | 0 |
| name168 | 3 | 1 | 1 | 34 | 0 |
| name169 | 2 | 1 | 0 | 32 | 0 |
| name170 | 3 | 1 | 0 | 32 | 0 |
